# WHO ANSWERS THE HOUSEHOLD FINANCIAL QUESTIONS: ANALYSIS OF STABILITY AND CHANGE

**DOI:** 10.1093/geroni/igz038.460

**Published:** 2019-11-08

**Authors:** Angela L Curl, Chido Mudzonga

**Affiliations:** Miami University, Oxford, Ohio, United States

## Abstract

There is limited research on who acts as household financial respondents (FR) for couples who are responding to surveys. The purpose of this study was to determine the amount of stability and change in FR, as well as predictors of FR status and change of FR status. Methodology: This study analyzed data from Health Retirement Study from 2006 to 2014. The sample comprised of 6,755 households of couples over the age of 50. Data were analyzed using Hierarchical Linear Modeling (HLM). Results: The results indicated that 18.13% households experienced one or more transitions of the financial respondent. Black non-Hispanics, males, those with higher education, earning a greater percent of the household’s income and those with higher cognitive ability were more likely to be the financial respondent than their counterparts. Higher depression (OR=1.22, p< .01) and lower cognitive ability (OR= 1.06, p<.01) were factors that predicted changes in the household’s financial respondent. Health (number of chronic conditions, self-rated health) and percentage of the household earnings are not significant in the change of financial respondent. Discussion: Change of respondent may result in differences in reporting of household financial resources in longitudinal studies. Changes in respondent may also be indicative of a more Egalitarian household, or serve as an early sign of changes in family dynamics or roles. As changes in mental health and cognitive ability may prompt a change of financial respondent, this highlights the importance of both spouses being financially literate and aware of the family’s economic resources, investments and obligations.

